# Testing Intergroup Threat Theory: Realistic and Symbolic Threats, Religiosity and Gender as Predictors of Prejudice

**DOI:** 10.5964/ejop.v14i2.1483

**Published:** 2018-06-19

**Authors:** Ana Makashvili, Irina Vardanashvili, Nino Javakhishvili

**Affiliations:** a The School of Arts and Sciences, Ilia State University, Tbilisi, Georgia; Department of Psychology, Webster University Geneva, Geneva, Switzerland; Heriot Watt University, Edinburgh, United Kingdom

**Keywords:** prejudice, intergroup threat theory, realistic and symbolic threats

## Abstract

The complex phenomenon of prejudice has been the focus of interest among social psychologists since the mid-20th century. The Intergroup Threat Theory (ITT) is one of the most efficient theoretical frameworks to identify the triggers of prejudice. In this study, using experimental design, we examined the effects of symbolic and realistic threats on prejudice that was measured by means of a modified social distance scale. The study participants were 611 undergraduate students from the country of Georgia. In addition to providing further support for ITT, the study showed that the level of religiosity moderated the effects between both types of threats and prejudice, although it had different indications for realistic and symbolic threats, while gender interacted only with symbolic threat. Implications of the findings are discussed.

In the age of growing political correctness, the Western social psychology has started to use terms such as subtle discrimination (Quillian, 2008), benevolent sexism (Glick & Fiske, 2002), barely perceptible racism (Aronson, 2012) and implicit prejudice (Oskamp & Schultz, 2005). Moreover, one of the main sources of anxiety toward an out-group is now believed to be not an irrational fear of the unknown, but rather the fear of presenting oneself in a negative light, that is, the desire to be seen as a non-prejudiced person (Dovidio & Gaertner, 2004). This means that while the blatant forms of hostility toward out-groups have weakened, the problem persists and has evolved to a degree that requires increasingly sophisticated measurement tools (Tetlock & Mitchell, 2008).

However, the Western social science developed particular interest in the problem at its explicit, even institutionalized, stage (Bogardus, 1925; Bogardus, 1938; Bogardus, 1958; Katz & Braly, 1935). In the United States, this happened in the 1920s, along with successive waves of non-protestant and Asian immigrants (Wark & Galliher, 2007), while in Europe (as well as in the United States) the interest in intergroup conflicts was especially intense in the mid-20^th^ century, in the wake of the dictatorships, the World War II and especially the Holocaust which sparked enormous shock and academic curiosity to understand what stood behind massive manifestations of prejudice, discrimination and intergroup conflicts (Hogg, 2006). More than half a century later, a recent influx of refugees from the Middle East and North Africa to the Western world has brought the issues of intergroup conflicts and threats to the attention of the public and governments (Foster, 2016) as the “refugee crisis” has been regarded as the largest-scale movement of people after the World War II (Smith, 2016). Moreover, it has been argued that the rhetoric used toward refugees is comparable with that used during the World War II (Tharoor, 2015). Thus, current events have become important incentives to resume studies in explicit prejudice and prejudice-related matters.

Whether prejudice is blatant or subtle depends heavily on the context. In the United States, for example, racial prejudice is highly condemned and equality is taught in schools (Boukari & Goura, 2012), while in Georgia, both the results of various nation-wide survey polls and real-time cases of homophobic, xenophobic and religious discrimination are to be qualified as the manifestation of explicit prejudice (Ramiah, Hewstone, Dovidio, & Penner, 2010). For example, the vast majority of the population surveyed thinks that people of other religious denominations should not enjoy the same rights as the Orthodox majority in Georgia (Sumbadze, 2012); media studies reveal the evidence of hate speech in national newspapers (United Nations Development Programme Georgia, 2013). The incident of May 17, 2013 in Tbilisi, Georgia can serve as an example of real-time discriminatory incidents, when a peaceful rally dedicated to the international day against homophobia was confronted by thousands of protestors opposing gay rights, who were allowed to break through the police cordon. Such attitudes and even discriminatory behaviors are widely supported, which validates Pettigrew's idea about one of the antecedents of prejudice – conformity (Pettigrew, 1958).

Prejudice is a psychological construct that can account for such negative attitudes and therefore, can be defined as “a hostile or negative attitude toward a distinguishable group on the basis of generalizations derived from faulty or incomplete information” (Aronson, 2012, p. 299).

For several decades, scholars have studied sources of prejudice including broad factors such as political, socio-economic (Greeley & Sheatsley, 1971) and socio-cultural contexts, the historical background and certain personality traits (Allport, 1954; Stephan, 2008) like social dominance orientation, right-wing authoritarianism and empathy (Dovidio et al., 2010; Javakhishvili, Beruashvili, & Kldiashvili, 2007; Laythe, Finkel, & Kirkpatrick, 2001; McFarland, 2010; Sidanius & Pratto, 1999; Stephan, 2008). Thus, it is difficult to attribute prejudice to a single factor or a single set of factors.

However, as Brewer (2007) argues, “the fact that individuals value, favor, and conform to their own membership groups (in-groups) over groups to which they do not belong (out-groups) is among the most well-established phenomena in social psychology” (p. 729). This phenomenon is the basis of Social Identity Theory (SIT; Tajfel & Turner, 1979). SIT posits that in-group members tend to look for negative aspects in out-groups, thereby improving their self-esteem. This, for its part, can lead to intergroup hostility and prejudice toward out-groups. In line with this reasoning, the Intergroup Threat Theory (ITT, [Stephan, Ybarra, & Morrison, 2009]) suggests that people are prone to anticipating threat from an out-group, which in turn fosters prejudice (e.g., Morrison et al., 2009; Myers, Abrams, Rosenthal, & Christian, 2013; Stephan et al., 2005).

The importance of threat and fear with regard to intergroup relations and prejudice has been pointed out by many authors (Adorno, Frenkel-Brunswik, Levinson, & Sanford, 1950; Allport, 1954; Levine & Campbell, 1972; Sherif, 1966; Smith, 1993) even before the development of ITT– a theory supported by a meta-analytical study of 95 samples (Riek, Mania, & Gaertner, 2006) demonstrating that perceptions of threat indeed trigger prejudice.

ITT authors (Stephan et al., 2009) stress that the theory is concerned with perceived rather than actual threats, as “perceived threats have real consequences, regardless of whether or not the perceptions of threat are accurate” (Stephan et al., 2009, p. 45). The study of attitudes toward immigrants in Germany, for example, found that the actual proportion of immigrants did not predict negative attitudes toward them, but the perceived proportion of immigrants did (Semyonov, Raijman, Tov, & Schmidt 2004). However, studies (e.g., Quillian, 1995) and historical experience (Aronson, 2012) also suggest that actual threats – poor economy, a large portion of minorities or immigrants – enhance negative attitudes toward out-groups which, according to Riek et al. (2006) means that national problems, including economic hardships, are ascribed to threat-inducing out-groups.

ITT distinguishes two types of perceived threats: realistic and symbolic threats. Perceived realistic threat, the concept that has its roots in the Realistic Group Conflict Theory (e.g. Sherif, 1966), is a threat to the actual – political, economic or physical – well-being (land, security, health, wealth, employment) of a group, while perceived symbolic threat is concerned with a group’s values, traditions, ideology, morals, and is expected to be more prominent when an in-group believes that their cultural values and traits are different from those of an out-group (Zárate, Garcia, Garza, & Hitlan 2004).

Whether perception of symbolic or realistic threat becomes salient depends upon the threat-invoking out-group (Stephan et al., 2009). Economically powerful out-groups or people with diseases might elicit realistic threats (Stephan et al., 2005), while socially marginalized out-groups, such as homosexuals (Haddock, Zanna, & Esses, 1993) and sects (Stephan et al., 2009), engender symbolic threats.

However, drawing a clear line between symbolic and realistic threats may be problematic in certain cases (Riek et al., 2006) as they may overlap. For example, symbolic threat posed by a religious out-group might involve realistic threat as well or evolve into the latter (Riek et al., 2006). The present study tries to address this issue by experimentally manipulating symbolic and realistic threats. Another reason to explore threats through experimental manipulation is the questionable validity of the threat instrument that has been used in most of the studies (e.g., Aberson & Gaffney, 2008; Dunwoody & McFarland, 2018; Myers et al., 2013; Stephan, Ybarra, Martinéz, Schwarzwald, & Tur-Kaspa, 1998; Stephan et al., 2002). Specifically, threats have often been measured by questionnaires including 12 (e.g., Stephan et al., 2002) or fewer (e.g., Dunwoody & McFarland, 2018) items such as “Blacks have more economic power than they deserve in this country” (in the case of realistic threat) and “Blacks and whites have different family values” (in the case of symbolic threat) (Stephan et al., 2002). These items raise questions regarding validity since they might be measuring prejudice already present in person rather than threat perceptions.

The need to experimentally study symbolic and realistic threats has also been emphasized by Riek, Mania, and Gaertner (2006) in their thorough review of studies on intergroup threats and negative attitudes, where the authors argue about certain methodological limitations: “One is the lack of experimental studies, especially in the domains of realistic and symbolic threats” (Riek et al., 2006, p. 346).

While it has been well established that perceived threats shape negative attitudes (e.g., Stephan et al., 2005), studies do not provide uniform results regarding associations between prejudice and demographic variables such as gender and the level of religiosity. A number of researchers (e.g., Altemeyer, 1998; McFarland, 2010; Parrillo & Donoghue, 2005) argue that males, as compared to females, are more prone to being prejudiced. However, the evidence is not consistent (see Herek, 2002; Hughes & Tuch, 2003). The findings also reveal associations between being religious and holding prejudices (e.g., Hall, Matz, & Wood, 2010; Rowatt, LaBouff, Johnson, Froese, & Tsang, 2009; Scheepers, Gijsberts, & Hello, 2002; Ugurlu, 2013), which can be either positive (e.g., Allport & Kramer, 1946; Hall et al., 2010) or negative (e.g., Laythe et al., 2001). Thus, further research is needed to better comprehend the role of gender and religiosity in prejudice.

At the same time, studies show cross-cultural differences in factors contributing to prejudice. For example, while race was identified as an important source of social distances among American students, religion and the employment status was found to contribute to negative attitudes in Greek and Japanese students, respectively (Triandis, Loh, & Levin, 1966). The study of the social distances of Georgian, German and Japanese students (Javakhishvili, Schneider, Makashvili, & Kochlashvili, 2012) found no significant effects of gender and religiosity on social distance scores in any of the three samples.

## Aims and Hypotheses

Considering the real-life relevance of threats (e.g., Quillian, 1995), the aim of the current study is to show that threat-evoking contexts elicit prejudice.

Drawing upon ITT, we test the effects of both, symbolic and realistic threats on prejudice and by manipulating the two threatening situations through exposing the participants to information about fictitious out-groups, we examine whether they have different effects as compared to the situation where threat is absent (control condition).

Furthermore, we explore the demographic variables that have been shown to be associated with prejudice. These variables include the participants’ gender and the level of religiosity. Expecting that both factors are related to prejudice, their interactions with threats are also tested to examine whether any of the two moderates the relation between threats and prejudice and whether the interaction is different for realistic and symbolic threats.

Given that symbolic threats are related to traditions, customs and values (Stephan et al., 2009), we assume that the interaction effect between religiousness and this type of threat will be stronger than for the realistic threat.

## Method

### Participants

The study participants were 611 undergraduate students from different universities of Tbilisi, Georgia. The majority of them, 95.1%, were Georgians, while others were ethnic minorities, including Armenians, Azerbaijanis, Russians, Ukrainians and Ossetians. To ensure homogeneity, we excluded data from non-Georgian participants, which reduced the total number of responses to 589. 92.2% of the remaining participants identified themselves as Orthodox Christians, 4.4% said they did not belong to any religious denomination, while 13 (3.4%) did not provide any response. The mean score of the participants' level of religiosity was 6.36 (2.407) with the minimum of 1 and the maximum of 10. Out of them, 166 (28%) were males and 423 (72%) were females, with the mean age of 20.37 (4.984), ranging from 18 to 25 years. The uneven gender distribution reflects the situation in the population of students from which the sample has been taken: this population consists of students from the humanities, social sciences and natural sciences, where females dramatically outnumber males. At the same time, the main aim of our design was to ensure equivalency between the three groups, which was met (see Table 1).

A test of homogeneity of variance was performed in order to check the equivalence of age distribution across experimental (symbolic and realistic threats) and control (“no threat”) conditions. Group mean (*p* = .193) and median (*p* = .680) comparisons showed no significant differences between the groups (see Table 1).

**Table 1:** Means and Standard Deviations for Age and Level of Religiosity Across Three Groups

Group	Age			Level of religiosity		
	*N*	*M*	*SD*	*N*	*M*	*SD*
Realistic threat	197	20.08	1.95	196	6.26	2.24
Symbolic threat	196	20.15	2.21	195	6.68	2.65
“No threat”	196	20.08	1.61	191	6.13	2.29

Similarly, a test of homogeneity of variance was used to look at the equivalence of distribution of the level of religiosity across the three groups. Again, no significant differences were found between experimental and control conditions based on the mean (*p* = .059) and median (see Table 1).

Crosstabs showed that gender distribution, although with predominantly more women in all the three groups, was still similar across the groups with 141 women versus 56 men in realistic threat condition, 131 women versus 65 men in symbolic threat condition and 151 women versus 45 men in control condition (“no threat).

Data collection took place between December 2015 to February 2017.

### Procedure

Before starting data collection, a detailed research project was submitted to the ethics committee of Ilia State University. Data collection started only after the ethical approval was obtained.

Having obtained approval from the universities, we entered the classes and asked the students if they wished to take part in the study of the role of mass media in a daily life. Those who agreed to participate were led to believe that the study was concerned with the daily coverage of events and the role of media in shaping public attitudes. They were asked to read a copy of a newspaper article and answer several questions, after which they were to fill out an eight-item attitude questionnaire about an out-group mentioned in the article and to provide demographic data. After completing all of the tasks, the participants were thoroughly debriefed: the true objectives of the study were disclosed and the students were given sufficient time to ask questions.

### Design

The study used a between-subjects factorial design with one factor, perceived threat, which was examined at three levels: realistic (*n* = 197) and symbolic threat (*n* = 196) in experimental conditions, and the absence of threat in the control condition (*n* = 196).

### Measures

#### Threat Manipulation

In order to manipulate realistic and symbolic threats, several vignettes were composed – a page length newspaper-type articles about a fictitious out-group called “Abirians” (invented solely for research purposes). The articles designed for experimental conditions (i.e., symbolic and realistic threats) contained information that the UN planned to settle in Georgia an ethnic group of Abirians, who currently resided in the Gaza Strip, and described potential threats of the plan for Georgia.

**a. Realistic threat manipulation:** in one version of the articles, the settlement of the Abirians in Georgia was associated with realistic threats: competition for jobs and higher education, increased taxes and the possible spread of diseases such as hepatitis C. An excerpt from the article read as follows:

As reported [by the organization], Abirians will immediately need workplaces on the local labor market upon their arrival in Georgia. According to the International Index of Education and Employability (IIEE), Abirians are quite similar to leading European nations. This means that they can easily compete with the local population in terms of both, education and labor market.

**b. Symbolic threat manipulation:** another version described symbolic threats and focused on the types of cultural differences which, according to a number of recent opinion polls, Georgians are particularly sensitive to: the out-group was reported to have a non-uniform religion, favor same sex marriage and adhere to such traditions and values that are clearly uncommon in Georgia. For example:

As reported [by the organization], Abirians are of the mixed race: a combination of the Caucasian and Asian races. As they reside at the crossroads of Muslim and Judaism cultures, they do not have a single religion: some of them are Muslims, some are the followers of Judaism, while others practice mixed Muslim and Jewish religious rites. However, according to the International Research Center of World Religions (IRCWR), the majority of them does not belong to any religion.

**c. Control condition:** the third version, designed for the control group (“no threat” condition), was similar in structure and style but described the out-group (Abirians) in neutral terms, with no references to threats whatsoever (it did not mention the prospects of the Abirians’ settlement in Georgia). For example:

As reported by PCW, the census of an ethnic group Abiri residing in Gaza Strip is already available. Historical sources suggest that the Abirians have been residing in the territory since the 1830s when Egypt conquered Palestine, and later, as a result of the British intervention, the Ottoman Empire regained control over the region.

To ensure that the participants read and comprehended the articles they were given several questions; for example, the Likert-scale items: “Please evaluate the style of the article. Did you find it objective?”

**Level of religiosity** was measured by a single 10-points scale item with 1 = *not at all religious* and 10 = *highly religious.*

**Prejudice** was measured by an updated version of the Social Distance Scale (Bogardus, 1925). Leaning upon the results of nation-wide survey polls (e.g., Caucasus Research Resource Centers, 2015; Sumbadze, 2012), human rights (Human Rights Watch, 2017) and media (United Nations Development Programme Georgia, 2013) reports and real-time incidents of homophobia, xenophobia and religious discrimination, which point to explicit forms of prejudice, Social Distance Scale was considered as an appropriate instrument. Based on our experience (Javakhishvili, 2005; Javakhishvili, 2011; Javakhishvili et al., 2012; Javakhishvili et al., 2016; Vardanashvili & Javakhishvili, 2016), the scale is a good measure of explicit prejudice as it is assumed to tap into the behavioral component of prejudice, versus, for instance, the feeling thermometer that tests the affective aspect of prejudice (Henry, 2008; Stefaniak & Bilewicz, 2016).

A revised version of the scale included eight 5-points Likert-scale items, e.g., “I would accept Abirians as my child’s teacher” or “I would make friends with Abirians,” with 1 = *strongly agree* and 5 = *strongly disagree*, higher scores indicating higher levels of social distance, and hence, prejudice, and lower scores indicating lower levels of prejudice.

The validity of the measure was examined using the confirmatory factor analysis (CFA) in Mplus (Muthén & Muthén, 2010). The first model considered one latent variable (social distance as a single construct) predicted by the respective eight items. The model did not fit the data. We then divided the scale into two parts, each representing a latent factor. The first one included three items, while the second factor covered the remaining five items.

The resulting model had a good fit, χ^2^(14, 589) = 20.38, CFI = .997, TLI = .993, RMSEA = .027, SRMR = .018. Therefore, the Social Distance Scale incorporated two factors. For the first factor the Cronbach’s Alpha was .829, while for the second factor it was .879. Consequently, two separate scores of the dependent variable for the first factor and the second factor were used in the further analysis. Data of factor loadings are provided in Table 2.

**Table 2:** Factor Loadings of the Confirmatory Factor Analysis (CFA) Two-Factor Solution for the Social Distance Scale (Revised)

Item	Factor 1	Factor 2
I would marry Abirians	0.63***	
I would accept Abirians as my child’s teacher	0.83***	
I would accept Abirians as my boss at work	0.82***	
I would make friends with Abirians		0.90***
I would accept Abirians as my neighbors		0.91***
I would sit next to Abirians in a public transport		0.73***
I would accept Abirians as my acquaintances		0.76***
I would accept Abirians in my country		0.66***

***p ≤ .001.

## Results

Descriptive statistics showed that threat manipulation had an effect on both factors of prejudice toward Abirians (see Table 3). The differences between the mean scores of prejudice across realistic threat and control ("no threat") condition as well as symbolic threat and control ("no threat") condition were verified by the one-way analysis of variance (ANOVA). No significant differences were found between the realistic and symbolic threat conditions. Mean scores of prejudice where higher for the first factor of prejudice, *F*(2, 586) = 25.45, *p* < .001 than for the second factor, *F*(2, 586) = 24.24, *p* < .001 (see Table 3).

**Table 3:** Means and Standard Deviations for Prejudice Toward Abirians

Group	The First Factor of Prejudice			The Second Factor of Prejudice		
	*N*	*M*	*SD*	*N*	*M*	*SD*
Realistic threats	197	3.39	0.96	197	2.07	0.76
Symbolic threat	196	3.47	1.14	196	2.22	0.97
“No threat”	196	2.82	0.85	196	1.68	0.62

The participants’ gender proved to be a significant variable only in the case of the second factor, *t*(584) = 3.36, *p* < .001. Male participants scored higher *M* = 2.167 (0.985) as compared to female participants, *M* = 1.92 (0.74).

The level of religiosity positively correlated with both forms of prejudice: a higher correlation was found for the first factor of prejudice, *r*(582) = .40, *p* < .001 as compared to the second factor, *r*(582) =.28, *p* < .001.

Since the level of religiosity was positively related to both factors of prejudice and gender had a significant effect on the second factor, simple regression was performed with threats and these variables as predictors.

Initially, we examined the predictors of the first factor of prejudice. The latter was entered as a dependent variable in the regression equation. Threat was turned into a dummy variable so that we were able to enter realistic and symbolic threats as predictors in Model 1. Both types of threats significantly contibuted to the variance of prejudice, *R^2^* = .08, *F*(2, 581) = 24.87, *p* < .001. The level of religiosity was added in Model 2, resulting in Δ*R^2^* of 15% with threats retaining significance and religiosity predicting prejudice, *R^2^* = .22, *F*(3, 581) = 46.68, *p* < .001. Results of regression are shown in Table 4.

**Table 4:** Regression on the First Factor of Prejudice

Predictor	β	
	Model 1	Model 2
Realistic threat	0.26***	0.25***
Symbolic threat	0.30***	0.26***
Level of religiosity		0.38***
*R^2^*	.08	.22
*ΔR^2^*		.15***

***p ≤ .001.

Likewise, we ran simple regression for the second factor of prejudice. Realistic and symbolic threats (turned into dummy variables) were entered in Model 1. Both of them again predicted prejudice, *R^2^* = .08, *F*(2, 580) = 24.01, *p* < .001. Gender (turned into a dummy variable) and the level of religiosity were added in Model 2. Again, threats predicted prejudice and gender and religiosity contributed significant variance with Δ*R^2^* of 9%, *R^2^* = .16, *F*(4, 580) = 27.95, *p* < .001. Regression results are shown in Table 5.

**Table 5:** Regression on the Second Factor of Prejudice

Predictor	β	
	Model 1	Model 2
Realistic threat	0.22***	0.21***
Symbolic threat	0.31***	0.26***
Level of religiosity		0.28***
Gender		0.14***
*R^2^*	.08	.16
*ΔR^2^*		.09***

***p ≤ .001.

22% of the variance of the first factor of prejudice was explained by symbolic and realistic threats and religiosity, while 16% of the variance of the second factor was explained by symbolic and realistic threats and religiosity plus gender. According to β-coefficients, for both, the first and the second factors, symbolic threat was a better predictor than realistic threat.

Since religiosity and gender contributed substantially to this association, we performed a moderation analysis by means of a special software Process, which is a supplement to SPSS (Hayes, 2013). This statistical technique uses regression to provide model outputs.

For the first factor of prejudice, the moderation of the effect of realistic and symbolic threats was tested by the level of religiosity, while for the second factor we examined the moderation of the effect of threats by the level of religiosity and gender. In total, four models were tested:

1. Realistic threat → Religiosity → The first factor of prejudice
2. Symbolic threat → Religiosity → The first factor of prejudice
3. Realistic threat → Religiosity → Gender → The second factor of prejudice
4. Symbolic threat → Religiosity → Gender → The second factor of prejudice

Model 1: Realistic threat → Religiosity → The first factor of prejudice. Overall, the model was significant: *F*(3, 578) = 47.12, *p* < .001, *R^2^* = .20. *b* coefficients are presented in Table 6.

**Table 6:** b Coefficients for the Model 1: Realistic Threat and Religiosity on the First Factor of Prejudice

Effect	*b*	*t*
Main and interaction effects of predictor variables		
Realistic threat	1.12***	4.71
Level of religiosity	0.21***	11.21
Realistic threat × Level of religiosity	-0.13***	-3.81
Conditional effect of realistic threat on the first factor of prejudice at 3 different values of the moderator – level of religiosity		
Level of religiosity 1 *SD* below mean - 3.95	0.58***	5.02
Level of religiosity at the mean - 6.36	0.26***	3.20
Level of religiosity 1 *SD* over mean - 8.77	0.06	-0.53

***p ≤ .001.

All of the effects – the main effects of realistic threat, the level of religiosity as well as their interaction – were significant, which indicates the moderating effect of religiosity. The most interesting information is provided in the second, lower part of the table: b coefficient decreased as the level of religiosity increased and, finally, it became nonsignificant (*p* = .595).

Model 2: Symbolic threat → Religiosity → The first factor of prejudice. Overall, the model was significant: *F*(3, 578) = 48.78, *p* < .001, *R^2^* = .20. *b* coefficients are presented in Table 7.

**Table 7:** b Coefficients for the Model 2: Symbolic Threat and Religiosity on the First Factor of Prejudice

Effect	*b*	*t*
Main and interaction effects of predictor variables		
Symbolic threat	-0.60**	-2.64
Level of religiosity	0.11***	5.36
Symbolic threat × Level of religiosity	0.14***	4.17
Conditional effect of symbolic threat on the first factor of prejudice at 3 different values of the moderator – level of religiosity		
Level of religiosity 1 *SD* below mean - 3.95	-0.06	-0.52
Level of religiosity at the mean - 6.36	0.27***	3.27
Level of religiosity 1 *SD* over mean - 8.77	0.59***	5.38

**p ≤ .01. ***p ≤ .001.

As in the case of realistic threat, religiosity moderated the association. Main effects of symbolic threat, the level of religiosity and their interaction were significant, although the direction of association was reversed: b coefficient and the level of religiosity increased simultaneously.

Model 3: Realistic threat → Religiosity → Gender → The second factor of prejudice. Overall, the model was significant: *F*(5, 575) = 18.28, *p* < .001, *R^2^* = .14. *b* coefficients are presented in Table 8.

**Table 8:** b Coefficients for the Model 3: Realistic Threat, Religiosity and Gender on the Second Factor of Prejudice

Effect	*b*	*t*
Main and interaction effects of predictor variables		
Realistic threat	0.89**	4.25
Level of religiosity	0.14**	8.68
Gender	0.32**	3.61
Realistic threat × Level of religiosity	-0.12**	-3.99
Realistic threat × Gender	-0.05	-0.31
Conditional effect of realistic threat on the second factor of prejudice at different values of the moderators – 3 levels of religiosity and two levels of gender		
Level of religiosity 1 *SD* below mean - 3.95 - for women	0.42**	3.81
Level of religiosity at the mean - 6.35 - for women	0.13	1.68
Level of religiosity 1 *SD* over mean - 8.76 - for women	0.15	-1.44
Level of religiosity 1 *SD* below mean - 3.95 - for men	0.37**	2.69
Level of religiosity at the mean - 6.35 - for men	0.09	0.68
Level of religiosity 1 *SD* over mean - 8.76 - for men	0.20	-1.27

**p ≤ .01.

For this model, only the level of religiosity proved to be a moderator in the association between realistic threat and the second factor of prejudice. All main and interaction effects were significant except the interaction of realistic threat and gender. For men as well as for women, effect of threat decreased in line with the increase of religiosity; in fact, it became nonsignificant.

Model 4: Symbolic threat → Religiosity → Gender → The second factor of prejudice. Overall, the model was significant: *F*(5, 575) = 24.37, *p* < .001, *R^2^* = .17. *b* coefficients are presented in Table 9.

**Table 9:** b Coefficients for the Model 4: Symbolic Threat, Religiosity and Gender on the Second Factor of Prejudice

Effect	*b*	*t*
Main and interaction effects of predictor variables		
Symbolic threat	-0.74***	-3.77
Level of religiosity	0.04*	2.23
Gender	0.16	1.73
Interaction of symbolic threat and level of religiosity	0.14***	5.30
Interaction of symbolic threat and gender	0.30*	2.05
Conditional effect of symbolic threat on the second factor of prejudice at different values of the moderators – 3 levels of religiosity and two levels of gender		
Level of religiosity 1 *SD* below mean - 3.95 for women	-0.18	-1.64
Level of religiosity at the mean - 6.35 - for women	0.16*	2.01
Level of religiosity 1 *SD* over mean - 8.76 - for women	0.50***	5.13
Level of religiosity 1 *SD* below mean - 3.945 - for men	0.12	0.89
Level of religiosity at the mean - 6.35 - for men	0.46***	3.83
Level of religiosity 1 *SD* over mean - 8.76 - for men	0.80***	5.73

*p ≤ .05. ***p ≤ .001.

The main effects of symbolic threat and the level of religiosity were significant, while that of gender was not. Both interactions were significant, which indicates the moderating effect of religiosity as well as gender. The effect of symbolic threat grew along with the level of religiosity both for men and women. However, effects of symbolic threat were stronger for men than for women.

## Discussion

The findings provide evidence that the manipulations of realistic and symbolic threats were successful and confirm that threats alone have a potential to account for an increase in prejudice, as has been shown by previous studies (e.g., Aberson & Gaffney, 2008; Riek et al., 2006; Stephan & Stephan, 1996; Stephan et al., 2002).

Additionally, along with realistic and symbolic threats, the level of religiosity significantly predicted both factors of prejudice, while gender emerged as a fourth predictor for the second factor.

The positive relationship between religiosity and prejudice has been shown in a number of studies (Allport & Kramer, 1946; Hall et al., 2010; Rowatt et al., 2009; Scheepers et al., 2002; Ugurlu, 2013), and as Gorsuch and Aleshire (1974) argue, religiousness is linked with higher levels of conventionality and lesser acceptance of people with different norms.

However, although a number of authors (e.g., Altemeyer, 1998; Parrillo & Donoghue, 2005; see McFarland, 2010) suggest that men, compared to women, are more prone to being prejudiced, the gender is not always positively associated with prejudice (see Herek, 2002; Hughes & Tuch, 2003; Javakhishvili et al., 2012). For example, Ekehammar, Akrami, and Araya (2003) experiments revealed that males scored higher on explicit prejudice measure, while women showed higher levels of implicit prejudice (Ekehammar et al., 2003). To some extent, the inconsistency of the evidence was reflected in our findings as gender predicted only the second factor of prejudice. Since the second factor implies less delicate relations with an out-group, the result might indicate that men and women tend to have similar attitudes when it comes to more delicate relations (the first factor of prejudice) with an out-group, while the distinction comes into play with less delicate (the second factor of prejudice) relations.

The moderational analysis showed that the interaction effect between realistic threat and religiosity was similar for both factors of prejudice. Religiosity moderated the association between realistic threat and prejudice: specifically, higher levels of religiosity were linked with a lower effect of realistic threat on both factors, which suggests that people with lower levels of religiosity tend to be less sensitive to perceiving realistic threat.

According to Cohrs and Ibler (2009), a moderational approach assumes that either threat perception might happen due to preexisting levels of certain personal dispositions (religiousness in this case) or that the dispositions “are activated from memory and rendered psychologically salient and influential by contextual features” (Cohrs & Ibler, 2009, p. 82) that correspond to the content of those personal dispositions. In line with this reasoning, our findings suggest that realistic threat (“contextual feature”) activates religiousness or that the preexisting levels of religiousness trigger perceptions of realistic threat.

The interaction effect between symbolic threat and level of religiosity was also similar for both factors of prejudice and, at the same time, opposite to that between religiosity and realistic threat: religiosity moderated the association between symbolic threat and prejudice however, higher level of religiosity was associated with a higher effect of symbolic threat on both factors. The finding has an implication for our hypothesis which proposed that the interaction effect between religiosity and symbolic threat would be stronger than that between religiosity and realistic threat. Although interaction coefficients were almost similar for realistic (*b* = - 0.13 for the first factor of prejudice and *b* = -0.12 for the second factor) and symbolic (*b* = 0.14 for both factors) threats, the finding suggests that the level of religiosity is more salient in case of symbolic threat perception, indicating the potential mediational chain between the variables (Baron & Kenny, 1986), which needs to be addressed in further analysis.

The results of the moderational analysis, altogether, correspond to the conceptualizations of the types of threats. Since symbolic threats include threats to traditions and values (Stephan et al., 2009), they are experienced on a more personal level and can be engendered by the out-groups that are perceived as having different traditions and values by people who embrace these values and traditions (Stephan, Ybarra, & Bachman, 1999). Religiosity which, in a broader sense can be regarded as an extension of traditions and values, tends to make people more vulnerable in the face of the out-group that adheres to different religious practices and norms. Indeed, according to Schwartz et al. (2012), traditionalistic values positively correlate with the importance of religion in one’s life.

On the other hand, realistic threats involve more rational responses in general, such as seeking information about an out-group and negotiating with it (Stephan et al., 2009), which might account for why religiosity – a less rational phenomenon positively related to likewise less pragmatic phenomena such as rigid morality, conventionality, the need for closure (see Jost, Glaser, Kruglanski, & Sulloway, 2003) and supernatural beliefs (Oliver & Wood, 2014) – takes over realistic threat in explaining prejudice.

The only association where gender had a moderational effect was that between symbolic threat and the second factor of prejudice: the effect of symbolic threat was stronger for men than for women, a finding which is somewhat controversial since realistic threats deal with competition over power and resources (Stephan et al., 2009), and males, in general, are more likely to compete over power. As Schwartz (2006) showed, being male is positively associated with power values. Furthermore, males tend to score higher on Social Dominance Orientation (SDO) (Sidanius & Pratto, 1999) which is a personal orientation supporting hierarchies and power dominance (Sidanius & Pratto, 1999). However, symbolic threat might have similar relevance to men in terms of power and resources.

Religiosity significantly contributing to prejudice has particular implications especially in the context of Georgia, where the majority (65.9%) of respondents surveyed identify themselves as veritable Christians rather than citizens of Georgia (Sumbadze, 2012), 90% of the population surveyed find religion important in their lives, more than half say they are religious (Caucasus Research Resource Centers, 2015) and 35% think that political opinions of priests and the congregation are important to them in making decisions when voting at parliamentary elections (National Democratic Institute, 2016), while the church, including the leader of the Georgian Orthodox Church, has explicitly expressed negative attitudes toward minorities, comparing gay people to drug addicts (Roth, 2013) and making a statement the day before a peaceful rally against homophobia (which was attacked by gay rights opposers led by priests) that the rally was “the violation of the majority rights, offence of their traditions, religion and their manner of thinking in general” (“Patriarqi xelisuflebisagan,” 2013).

Moreover, according to the nationwide poll (Caucasus Research Resource Centers, 2015), religion in everyday lives of young population aged 18-35 is as important (90%) as to older generations (91% for the age group of 36-55 and 89% for people above 56). Indeed, “the vast majority of religiously active population are young people, living in the capital city, with high education, who spent their childhood in the 1990s, in the most harsh (insecure) political and economic situation” (Kekelia, Gavashelishvili, Ladaria, & SulkhanishvilI, 2013, p. 70).

While an insecure or, in other words, threat-arousing environment might give rise to religious sentiments (e.g., Greenberg, Pyszczynski, & Solomon, 1986; Kekelia et al., 2013), the opposite can also be true: religious sentiments might give rise to threat perceptions (Cohrs & Ibler, 2009). However, in both cases, as our results have demonstrated, threats gain importance in shaping prejudice – a finding which, again, is particularly relevant for Georgia, where the population tends to face instability, while unemployment, economic crisis and territorial integrity (one-third of the Georgian territory is occupied by Russia) are perceived to be top three concerns (International Research Institute, 2017). Given the context, people are more prone to experience threats from out-groups, which leads to prejudice (Aronson, 2012; Quillian, 1995). Real-life incidents of explicit prejudice indeed fit into this reasoning. The examples include religious discrimination having taken place in one of the Georgian villages when the Orthodox Christians attacked local Muslim population to hinder their religious service (“Axali detalebi,” 2012) or, to cite a more recent example, the rally organized by the so-called Georgian March occupied the capital streets, overtly expressing ethnic discrimination and stating that their goal was to “clear Georgia from illegal immigrants” (Gvarishvili, 2017).

Finally, the present study provides further support for ITT, demonstrating that both, realistic and symbolic threats account for prejudice, while the level of religiosity and being male (only in case of the second factor of prejudice) are additional contributors.

One of the most obvious limitations of the study is the specific age-group of the sample, which prevents generalization to other age groups. This also leads to the question of external validity: the fictitious nature of the threat-inducing out-group in the current study raises a concern whether the findings can be inferred to similar situations in real-life.

Further analysis is needed to identify the mechanism of how the variables in question are ordered in explaining prejudice. The mediational analysis can clarify whether the association between symbolic threat and prejudice is mediated by the level of religiosity or if symbolic threat itself is a mediator.

Additionally, as the relationship between religiosity and prejudice is not always positive and uniform (e.g., Herek, 1987; Laythe et al., 2001), suggesting that the concept includes more than one dimensions (e.g., Laythe et al., 2001) either positively or negatively related to prejudice, it would be more informative to measure these dimensions and gain a better insight in the concept, as well as to determine which dimensions of religiosity predicts prejudice and how the latter is connected to other well-established predictors, such as right-wing authoritarianism and SDO (McFarland, 2010). The role of gender also needs further research and might be better understood in case of a more balanced distribution.
